# Supplementary material for: Novel prognostic biomarkers of pouchitis after ileal pouch-anal anastomosis for ulcerative colitis: Neutrophil-to-lymphocyte ratio
Source: PLoS One. 2020 Oct 26;15(10):e0241322. doi: 10.1371/journal.pone.0241322 (PMC7588073; doi:10.1371/journal.pone.0241322)
Supplement: Attachment — Submitted filename: response_to_reviewersR1003.docx [file pone.0241322.s003.docx]

**October 5^th^, 2020**

**Dear Prof. Emiko Mizoguchi,**

**Academic Editor**

**PLOS ONE**

Dear Editor:

We appreciate the opportunity to submit a revised version of our manuscript, titled **“Novel prognostic biomarkers of pouchitis after ileal pouch-anal anastomosis for ulcerative colitis: Neutrophil-to-lymphocyte ratio”** (manuscript ID; PONE-D-20-18412), for publication in *PLOS ONE* as an original article. We believe that we have addressed all comments raised by the reviewers, as detailed in the accompanying point-by-point reply.

We have also reanalyzed the data and revised the manuscript and all the tables and figures with the cooperation of Satoko Ohfuji, who is a statistic expert in the Department of Public Health at our university. We have added her as a co-author. She declares that she has no conflicts of interest.

We hope that these changes support and enhance our message: NLR may be a novel and useful indicator for predicting the development of pouchitis after ileal pouch-anal anastomosis in patients with ulcerative colitis.

The authors declare the following:

1. All authors concur with the submission of this revised manuscript.
2. This manuscript has not been published elsewhere in part or in its entirety and is not under consideration by another journal. This study design was approved by Osaka City University Hospital Certified Review Board, according to the Declaration of Helsinki. All the authors contributed to the manuscript and agreed with this submission to your journal.
3. I have read the journal’s policy and the following authors of this manuscript have a competing interest: All authors declare that they have no conflicts of interest and that there were no external funding sources for this study. This does not alter our adherence to *PLOS ONE* policies on sharing data and materials.

Many thanks for the opportunity to revise our manuscript. We hope that this manuscript is now acceptable for publication in *PLOS ONE*.

Sincerely yours,

Shuhei Hosomi, M.D., Ph.D.

Department of Gastroenterology, Osaka City University Graduate School of Medicine

1-4-3, Asahi-machi, Abeno-ku, Osaka, 545-8585, Japan.

Tel.: +81 6 6645 3811; fax: +81 6 6645 3813.

E-mail: m1265271@med.osaka-cu.ac.jp

**Response to the Reviewer #1 comments:**

We appreciate your constructive comments and valuable suggestions as they helped us to revise and improve this manuscript. As indicated in the responses that follow, we have considered these comments and suggestions in the revised version of our manuscript. All changes in the manuscript in response to the critiques are indicated with yellow highlights.

*1. I could not accept this result. The authors need to exclude the one-stage surgery data in the results if the authors decide to use postoperative data. Or the authors need to increase the number of patients to show a significant difference using pre-operative data if the authors include the one-stage surgery data.*

We are in full agreement that we should have excluded the patients with one-stage surgery in the analysis. Among 50 patients included in this study, one-stage surgery was performed for only one patient. We excluded this patient and recalculated the data. The results did not change significantly. We revised all Figures and Tables and changed it to the recalculated value in the manuscript.

*2. The authors need to prove that the number of patients is statistically sufficient in this study.*

We are in full agreement that we should have mentioned whether the sample size is sufficient because the sample size in this study was rather small. We calculated the required sample size with the cooperation of Dr. Satoko Ohfuji, who is a statistic expert in the Department of Public Health at our university. Sample size calculation as a post-hoc analysis indicated that a total of 38 patients were required to detect a significant association between high NHR and the development of pouchitis with the following assumption: an α level of 0.05, a β level of 0.20, half of the patients were allocated to the high NLR group, the incidences of pouchitis in patients with high and low NLR group were 54% and 19%, registration period was 13 years, and the follow-up period was 1.5 years. Therefore, the sample size in this study was satisfied to examine the association between NLR and the development of pouchitis. We have added the following sentences in the Discussion. We have also added Dr. Satoko Ohfuji as a co-author.

“As the sample size of this study was relatively small, we calculated the required sample size. Sample size calculation as a post-hoc analysis indicated that a total of 38 patients were required to detect a significant association between high NHR and the development of pouchitis with the following assumptions: an α level of 0.05, a β level of 0.20, half of the patients were allocated to the high NLR group, the incidences of pouchitis in patients with high and low NLR group were 54% and 19%, registration period was 13 years, and the follow-up period was 1.5 years. Therefore, the sample size in this study was satisfied to examine the association between NLR and the development of pouchitis.” (lines 233-239)

*3. Why did the authors consider only TNF antibody as a background factor? There is a high possibility that steroids and immunomodulators are gradually being discontinued after surgery, so I think that it is necessary to consider them as background factors.*

We appreciate your valuable suggestion. Several studies have reported the risk factors for pouchitis. Regarding medications before the operation, only a total steroid dose of > 10000 mg [Koike Y, et al. The Journal of surgical research. 2019; 238: 72-8.] and use of infliximab [Mor IJ, et al. Diseases of the colon and rectum. 2008; 51: 1202-7.] were reported to be risk factors for pouchitis, to the best of our knowledge. In this study, we focused only on anti-TNF-α antibody therapy, as we could not calculate the total steroid dose for some cases. We should have mentioned the influence of other medications on the development of pouchitis. Although the use of immunomodulators, corticosteroids, anti-TNF-α antibody therapy, and calcineurin inhibitor therapy was not identified as a risk factor for pouchitis, we could not say that these medications were not associated with the development of pouchitis. This is because there are several biases, such as drug discontinuation during the course and the sample size was relatively small. We rephrased the manuscripts as follows: “ Regarding medications before the operation, only a total steroid dose of > 10000 mg [13] and the use of infliximab [14] was reported to be a risk factor for pouchitis, to the best of our knowledge. In this study, although the use of immunomodulators, corticosteroids, anti-TNF-α antibody therapy, and calcineurin inhibitor therapy was not identified a risk factor for pouchitis, we could not say that these medications were not associated with the development of pouchitis because of the relatively small sample size and several biases such as drug discontinuation during the course.” (lines 227-232)

*4. I agreed that the number of CD19+CD138+ cells, which are an immature subset of IgG-producing plasma cells, was significantly increased in the peripheral blood and inflamed colon of patients with active UC. However, I could not agree that a relatively low percentage of lymphocytes in peripheral. The authors need to show whether CD19+CD138+ cell subset is identified as a highly fluorescent population by an XE-5000 hematology analyzer. Or the authors try to explain why the lymphocytes are reduced in detail.*

We are in full agreement that we should have mentioned the details of morphological features of the CD19+CD138+ cell subset. As shown in our department’s previous study, immunoelectron microscopy clearly showed that the CD19+CD138+ cells had a feature of plasma cells; eccentrically located nuclei and abundant rough endoplasmic reticula [Jinno Y. et al. Virchows Archiv 2006;448(4):412-21]. Therefore, this cell population could be identified as a highly fluorescent population distinct from lymphocytes by hematology analyzer, implying that UC patients with abundant CD19+CD138+ cells would have a relatively low percentage of lymphocytes in peripheral blood. We discussed this mechanism as one of the possible mechanisms to explain how NLR could predict the development of pouchitis. We rephrased the manuscript as follows:

“This cell subset was reported to have a feature of plasma cells; eccentrically located nuclei and abundant rough endoplasmic reticula, by immunoelectron microscopy [39]. Therefore, this cell population could be identified as a highly fluorescent population distinct from lymphocytes by a hematology analyzer, implying that UC patients with abundant CD19+CD138+ cells would have a relatively low percentage of lymphocytes in peripheral blood. This might be one of the possible mechanisms to explain how the NLR could predict the development of pouchitis.” (lines 210-215)

**Response to the Reviewer #2 comments:**

We appreciate your constructive comments and valuable suggestions that have helped improve this manuscript. As indicated in the responses that follow, we have considered these comments and suggestions, which are reflected in our revised manuscript. All changes in the manuscript made in response to the comments are highlighted in yellow.

1. *The reviewer#1 mentioned the continued use of corticosteroids, immunomodulators, calcineurin inhibitors, and anti-TNF therapy during the postoperative course.* *Because, there are several biases as drug discontinuation during the course. However, the authors have not addressed all of them in this manuscript. The authors would be better to analyze anti-TNF therapy as well as other therapies as background factors and briefly mentioned in the manuscript.*

We are in full agreement that we should have mentioned other medications. As mentioned above, in this study, although the use of immunomodulators, corticosteroids, anti-TNF-α antibody therapy, and calcineurin inhibitor therapy was not identified as a risk factor for pouchitis, we could not say that these medications were not associated with the development of pouchitis because there are several biases such as drug discontinuation during the course and the sample size was relatively small. We rephrased the manuscript as follows: “Regarding medications before the operation, only a total steroid dose of > 10000 mg [13] and the use of infliximab [14] was reported to be a risk factor for pouchitis, to the best of our knowledge. In this study, although the use of immunomodulators, corticosteroids, anti-TNF-α antibody therapy, and calcineurin inhibitor therapy was not identified a risk factor for pouchitis, we could not say that these medications were not associated with the development of pouchitis because of the relatively small sample size and several biases such as drug discontinuation during the course.” (lines 227-232)
